# How good is a simple MRI score for predicting cerebral palsy and more?

**DOI:** 10.1111/dmcn.70245

**Published:** 2026-03-19

**Authors:** Linda S de Vries, Frances M Cowan

**Affiliations:** ^1^ Department of Pediatrics, Division of Neonatology Leiden University Medical Center Leiden the Netherlands; ^2^ Department of Paediatrics Imperial College London UK

## Abstract

**This commentary is on the original article by Dathe et al. on pages** 1729‐1739 **of this issue.**

Brain magnetic resonance imaging (MRI) is often used at term‐equivalent‐age (TEA) in high‐risk infants born preterm. In the study by Dathe et al., the authors assess MRI obtained at TEA using their Total Abnormality Score (TAS) with the aim of predicting the development of cerebral palsy (CP) by 24 months of age.^1^ The infants in the study were born preterm, at 32 + 0 weeks' gestation or less and/or weighing less than 1500 g. This group first reported on the TAS in 2018, a scoring system based on the Kidokoro score with a few modifications.^2^

In the present study, nine of 137 infants developed CP and they report a good sensitivity of 88.9% and specificity of 91.4% using a cut‐off value of 9.5 on the TAS, predicting CP in eight of nine infants. However, in all, 19 infants had a score ≥9.5, 11 of whom did not develop CP, resulting in a low positive predictive value of 42%. Interestingly, infants with higher TAS scores who did not develop CP had significantly lower cognitive and motor composite scores, and gross motor standard scores on the Bayley Scales of Infant and Toddler Development, Third Edition.

There are questions regarding changes the authors made when developing the TAS compared to the original Kidokoro score, and also about a few items that are different from their original publication on the TAS. For example, they no longer measure ventricular size but now use the word ‘obvious’ to describe ventricular enlargement. Would it not be more robust to continue ventricular measurements which are easy to do? Another difference is that for the worst white matter injury (WMI) score, they now use ‘cystic lesions in the periventricular white matter (without evidence of haemorrhage) or a cluster of ≥6 punctate lesions’, as if these are equivalent, while they previously restricted the grade 4 WMI to cystic periventricular lesions. We think this former approach is preferable as only 10% to 20% of the infants with punctate white matter lesions (PWMLs) develop CP, usually level I to II on the Gross Motor Function Classification System. Although restricting PWMLs to the cluster type could result in a slightly higher percentage of infants developing CP, it certainly would not be as high as for cystic WMI.^3^ Besides the number of PWMLs, their site is important, assessed either using the ‘simple white matter imaging rule’ or whether they cross the central sulcus in a sagittal plane.^4^

Finally, myelination was scored differently from the Kidokoro score, with the expectation that myelination would be seen in the optic radiation at TEA. In most infants at TEA, supratentorial myelination is restricted to the posterior limb of the internal capsule and corona radiata, and not seen in the optic radiation until 2 to 3 months post‐term age.

The study focuses on the TAS score for the TEA MRI. Of the nine infants who developed CP, four had severe intraventricular haemorrhage and five had cystic periventricular leukomalacia. It is likely, but not mentioned, that these overt brain lesions would already have been detected with serial cranial ultrasound.

Several publications have shown that meeting two of three criteria (MRI abnormalities; absence of fidgety movements; Hammersmith Infant Neurological Examination score below age‐appropriate cut‐off values) has an accuracy of 90% for predicting CP.^5^ It would have been of interest to see whether combining the TAS with one or two of these other criteria would reduce the false positive rate.

Whilst this simplified MRI scoring system seems appealing, more than 50% of infants with a score above their cut‐off value did not develop CP. It might be more accurate to say that this simple score is helpful in identifying infants with poorer cognitive and motor outcomes, rather than being an accurate predictor of CP.

## Data Availability

Not required.
